# Energy-Resolved Mass Spectrometry and Mid-Infrared Spectroscopy for Purity Assessment of a Synthetic Peptide Cyclised by Intramolecular Huisgen Click Chemistry

**DOI:** 10.3390/mps7060097

**Published:** 2024-12-02

**Authors:** Alicia Maroto, Ricard Boqué, Dany Jeanne Dit Fouque, Antony Memboeuf

**Affiliations:** 1 Univ Brest, CEMCA, CNRS, UMR 6521, 29238 Brest, France; 2 Department of Analytical Chemistry and Organic Chemistry, Universitat Rovira i Virgili, C/Marcel⋅lí Domingo 1, 43007 Tarragona, Spain

**Keywords:** peptide isomers, quantification, click chemistry, energy-resolved mass spectrometry, infrared microscopy, multivariate and univariate calibration, cyclic peptide

## Abstract

Cyclic peptides have higher stability and better properties as therapeutic agents than their linear peptide analogues. Consequently, intramolecular click chemistry is becoming an increasingly popular method for the synthesis of cyclic peptides from their isomeric linear peptides. However, assessing the purity of these cyclic peptides by mass spectrometry is a significant challenge, as the linear and cyclic peptides have identical masses. In this paper, we have evaluated the analytical capabilities of energy-resolved mass spectrometry (ER MS) and mid-infrared microscopy (IR) to address this challenge. On the one hand, mixtures of both peptides were subjected to collision-induced dissociation tandem mass spectrometry (CID MS/MS) experiments in an ion trap mass spectrometer at several excitation energies. Two different calibration models were used: a univariate model (at a single excitation voltage) and a multivariate model (using multiple excitation voltages). The multivariate model demonstrated slightly enhanced analytical performance, which can be attributed to more effective signal averaging when multiple excitation voltages are considered. On the other hand, IR microscopy was used for the quantification of the relative amount of linear peptide. This was achieved through univariate calibration, based on the absorbance of an alkyne band specific to the linear peptide, and through Partial Least Squares (PLS) multivariate calibration. The PLS calibration model demonstrated superior performance in comparison to univariate calibration, indicating that consideration of the full IR spectrum is preferable to focusing on the specific peak of the linear peptide. The advantage of IR microscopy is that it is linear across the entire working interval, from linear peptide molar ratios of 0 (equivalent to pure cyclic peptide) up to 1 (pure linear peptide). In contrast, the ER MS calibration models exhibited linearity only up to 0.3 linear peptide molar ratio. However, ER MS showed better performances in terms of the limit of detection, intermediate precision and the root-mean-square-error of calibration. Therefore, ER MS is the optimal choice for the detection and quantification of the lowest relative amounts of linear peptides.

## 1. Introduction

Cyclic peptides are commonly found in nature, where they play essential roles in biological processes. The cyclic structure confers several advantages, including increased proteolytic stability, reduced toxicity, target specificity and higher membrane permeability. Due to these properties, cyclic peptides have become an important area of research, particularly in drug discovery, where they are employed in the design of therapies for a range of diseases. To date, more than 40 cyclic peptides have been employed for the treatment of cancer, metabolic disorders and infectious diseases [1,2,3,4].

Various synthetic methods can be employed to cyclise linear peptides, including disulfide cyclisation, macrolactamisation, thiol alkylation and “click chemistry” via the Huisgen reaction [2,5,6]. The Huisgen reaction is a copper-catalysed cycloaddition between an azide and an alkyne, resulting in the formation of a triazole. This reaction has the advantage of being compatible with numerous functional groups. Moreover, incorporating the triazole moiety into the peptide structure enhances rigidity and provides resistance to protease degradation [2,7,8]. Nevertheless, as no atom is lost in this “click” reaction, the cyclic peptide and its linear precursor are isomers with the same molecular mass, making them indistinguishable by single-stage mass spectrometry.

Tandem mass spectrometry can easily distinguish between two isomers, provided that each isomer produces unique fragment ions specific to that isomer [9,10]. Nevertheless, several studies on synthetic polymers and peptides cyclised with the Huisgen reaction have demonstrated that the most significant fragment ions obtained from the cyclic form and its linear precursor are also isomers and originate from the loss of N_2_, either from the triazole group or from the azide moiety [11,12,13]. Consequently, the detection of residual linear precursor in the sample of cyclised products is not feasible through the simple visual inspection of MS/MS spectra. This renders the usual quantification methods, such as MRM, inapplicable for Huisgen cyclised peptides. Therefore, alternative analytical strategies must be employed to assess the purity of these cyclic peptides and the absence of their isomeric linear precursors.

Several strategies have been proposed to differentiate isomers by mass spectrometry, including the kinetic method [14,15,16,17], Ion Mobility Mass Spectrometry [18,19,20,21,22] and energy-resolved mass spectrometry (ER MS) [12,13,17,23,24,25,26,27,28,29]. In ER MS, collision-induced dissociation (CID) experiments are performed to obtain MS/MS spectra at multiple excitation voltages. In this context, Bartolucci et al. have successfully employed ER MS data to quantify co-eluted isomers by multilinear regression [17,23,24,25,26]. Complementing this approach, Memboeuf et al. have shown that ER MS, and, in particular, the Survival Yield (SY) plots, can be used for structural analysis of isobaric compounds [30] to quantify isomeric and isobaric mixtures [12,13,27] and to remove isobaric interferences in liquid chromatography [31,32]. The SY curves of isomeric mixtures can be expressed as a linear combination of the SY curves of the pure isomeric compounds [13,30]. Typically, the SY curves of isomeric mixtures will lie between the SY curves of both pure compounds, with their position being related to the relative concentration of each isomer in the mixture. Subsequently, linear and multivariate calibration models can be calculated to establish the relationship between the SY and the relative amount of both isomers in the sample mixtures [13]. In this context, we have previously demonstrated that this method can be employed to assess the purity of a Huisgen-cyclised polylactic polymer [11,12] as well as a Huisgen-cyclised peptide [13].

Fourier Transform Infrared (FT-IR) microscopy represents a rapid alternative to traditional analytical techniques [33]. One of the key advantages of this technique is that it requires only a small amount of sample, minimal sample preparation and allows for the examination of small spatial regions, providing molecular-level information, such as functional groups, bonding types and molecular conformations. The distribution of chemical entities can be investigated based on specific marker bands [34]. A wide range of biological investigations have been conducted by FT-IR microspectroscopy, including cancer detection [35], plant tissue analysis [36] and conformational studies of proteins and peptides [37], among others. The combination of advances in IR instrumentation with the application of chemometric tools has also rendered this technology an optimal choice for the rapid screening of a wide range of analytes in food analysis. In this context, it has been successfully applied to detect acrylamide in potato chips [38] and milk adulteration [39]. To the best of our knowledge, FT-IR microscopy has not yet been applied to assess the purity of peptides cyclised with the Huisgen reaction.

The objective of this paper is to evaluate the analytical capabilities of energy-resolved mass spectrometry and mid-infrared microscopy for the detection and quantification of a linear isomeric peptide present in a sample of a cyclic peptide synthesised with the Huisgen reaction. To this end, a model linear peptide with the sequence LAIFPWFLHPVAIGHA was synthesised and cyclised with the Huisgen reaction to obtain its isomeric cyclic peptide. Subsequently, both isomeric peptides, as well as their mixtures, were analysed by ER MS and IR microscopy. The relative amount of linear peptide was calculated by univariate and multivariate calibration. The analytical performance of IR microscopy was then compared with that of energy-resolved mass spectrometry in terms of detection limits, precision, the root-mean-square-error of calibration and the linearity interval.

## 2. Materials and Methods

### 2.1. Chemicals and Sample Preparation

**Figure 1:**
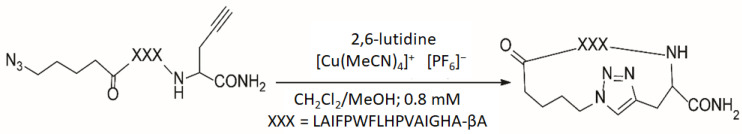
Synthesis of the cyclic peptide from the linear peptide by “click chemistry” using the Huisgen reaction.

Methanol (HPLC-MS grade) and caesium chloride (purity > 98%) were purchased from Sigma-Aldrich. The linear and cyclic peptides were synthesised at the Nanobio Platform (Université Grenoble Alpes, Grenoble, France). The structures of the two peptides are shown in Figure 1. The linear peptide precursor (2007.2 Da) contains a basic histidine residue and has the following structure X-LAIFPWFLHPVAIGHA-βA-Z (where X is the azide moiety, βA is the beta-alanine amino acid and Z is the group with the alkyne function). The linear peptide was assembled on a Syro II peptide synthesiser using the same Fmoc strategy as previously described [13].

Separate stock solutions of the linear and cyclic peptides were prepared at 50 μM in water/methanol (1:1). Caesium chloride was added to all solutions at 180 μM to form the caesium adducts of the peptides. Calibration standards were prepared in water/methanol (1:1). The total concentration of linear and cyclic peptides was 5 μM. The mixtures were prepared with the following linear/cyclic peptide ratios: 1:0; 0.5:9.5; 1:9; 1.5:8.5; 2:8; 3:7; 4:6; 5:5, 6:4 and 0:1.

The samples measured by IR microscopy were prepared at a total concentration of 5–10^−4^ M in water/methanol (1:1). In this case, CsCl was not added to the samples. The mixtures were prepared with the following linear/cyclic peptide ratios: 0:1; 0.5:9.5; 1:9; 1.5:8.5; 2:8; 3:7; 4:6; 5:5; 6:4; 8:2 and 1:0. All stock and working solutions were stored at −20 °C until use.

### 2.2. Energy Resolved Mass Spectrometry

Mass spectrometry experiments were performed in positive ion mode using an HCTplus ion trap mass spectrometer (Bruker Daltonics, Bremen, Germany) equipped with an Agilent Technologies electrospray ionisation (ESI) source. Solutions were introduced by direct injection using a syringe pump at a flow rate of 2 µL/min^−1^. The nebulising gas (N_2_) pressure was maintained at 10 psi, and the drying gas flow rate was set at 5 L-min^−1^, heated to 300 °C. Helium was used as the trapping and collision gas at a pressure of 3.00 × 10^−5^ mbar. Instrument parameters included a capillary voltage of 3.8 kV, an end plate voltage of −0.5 kV, a skimmer voltage of 40 V and a trap drive of 110.

For MS/MS spectra acquisition, precursor ions were isolated using a 1 *m*/*z* isolation window followed by a fragmentation delay of 200 ms, a fragmentation time of 100 ms and a fragmentation width of 10 *m*/*z*. Spectra were recorded for 1 min at each excitation voltage, and data from the final 45 s of each acquisition were combined to enhance statistical reliability and reproducibility.

Data acquisition and mass spectra processing were performed using DataAnalysis 3.3 software (Bruker Daltonics, Bremen, Germany) with default parameters including background reduction, smoothing and peak centring. Survival yield (SY) curves were generated using the LibreOffice 24.8.3 software package (freely available) [40] and plotted using SciDAVis 2.7 software [41]. Fitting of SY curves was performed using the Boltzmann model available in SciDAVis.

### 2.3. Infrared Microscopy

A ThermoFisher Scientific Nicolet iN10 MX microscope was used to obtain mid-infrared (675–4000 cm^−1^) spectra by specular reflectance. Background noise was recorded before each acquisition and subtracted from the spectra. The spectra of each peptide mixture were obtained by creating a grid of 800 µm by 600 µm. The grid contains 100 evenly spaced points. The number of scans recorded for each point was 16, and the spectral resolution was 4 cm^−1^. During acquisition, the Omnic Picta 1.7.190 software (Thermo Fisher Scientific, Waltham, MA, USA) averaged the 16 scans and provided 1 average IR spectrum per point. A total of 100 spectra were collected per mixture. The total acquisition time was 50 min per sample. Three replicates were performed for each mixture. Reflective aluminium-coated slides with 12 numbered spots were used to facilitate identification of the peptide mixture during IR acquisition. A total of 2 µL of the peptide mixture at 5 × 10^−4^ M was added to the spot. Preprocessing of IR spectra and multivariate calibration models were performed with Matlab R2024a (The Mathworks, Inc., Natick, MA, USA) and the PLS_Toolbox 9.3.1 (2024) (Eigenvector Research, Inc., Manson, WA, USA).

## 3. Results and Discussion

### 3.1. Energy-Resolved Mass Spectrometry and the Survival Yield Technique

**Figure 2:**
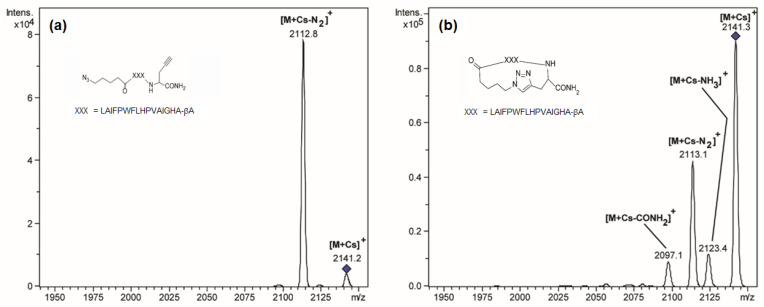
MS/MS spectra of linear (**a**) and cyclic (**b**) caesium-cationised peptides at 2.5 V excitation voltage. No peaks were observed outside of the mass range displayed in these MS/MS spectra.

ER MS experiments were performed by measuring the MS/MS spectra of the caesium-cationised peptides at different excitation voltages ranging from 1.7 V to 2.9 V. Figure 2 shows the MS/MS spectra of the cyclic and linear peptides at 2.5 V. At this excitation voltage the linear peptide is almost completely fragmented, whereas the cyclic peptide is slightly fragmented, and the precursor ion peak (at 2141.3 *m*/*z*) has the higher intensity. The major fragment of the linear peptide corresponds to the loss of N_2_ (at 2112.8 *m*/*z*). This fragment is also observed for the cyclic peptide. As there are no specific fragments for the linear peptide, it is not possible to detect the presence of the linear peptide by visual inspection of the MS/MS spectra of the cyclic peptide samples. However, the difference in excitation energy required to fragment both peptides can be used to detect the presence of linear peptides. In this sense, energy-resolved mass spectrometry and, more specifically, the Survival Yield (SY) were used to detect and quantify the relative amount of linear peptide.

**Figure 3:**
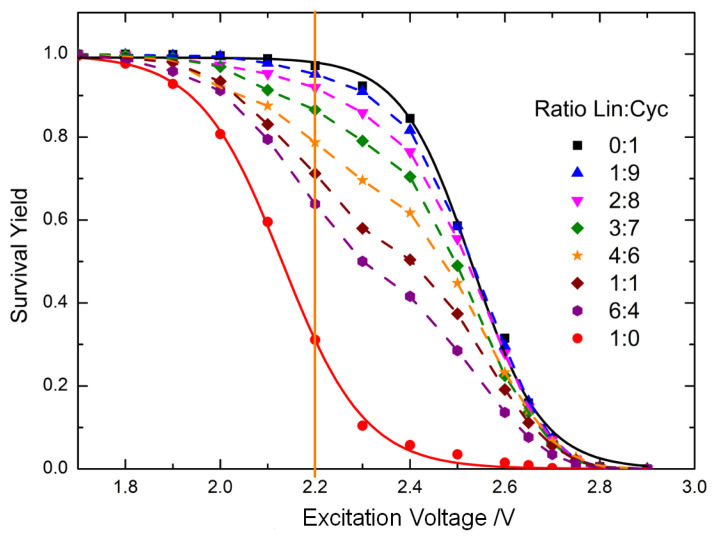
Survival Yield (SY) curves of mixtures of linear and cyclic peptides at different molar ratios. The SY is calculated from the MS/MS spectra of the caesium-cationised peptides. The SY of the mixtures lies between the SY curves of the linear and cyclic peptides. The orange vertical line corresponds to the excitation voltage at which the univariate calibration model was calculated to relate the SY to the molar ratio of the linear peptide.

The Survival Yield (SY) was calculated at each excitation voltage as the ratio of the precursor ions peak intensity and the Total Ion Current (TIC) [11,12,13,28,29,30,31].

(1) $$SY=\frac{I_{precursor}}{I_{precursor}+\sum I_{fragment}}$$

where *I_precursor_* is the intensity of the precursor ions peak, and *I_fragment_* is the intensity of each fragment ions peak obtained from the MS/MS experiment. SY curves were then obtained by plotting SY against the excitation voltage (see Figure 3).

Figure 3 shows the SY curves of the pure cyclic and linear caesium-cationised peptides, which are sigmoidal. The SY curve of the cyclic peptide shows a significant shift towards higher excitation voltages compared to that of the linear peptide. This is consistent with the MS/MS spectra in Figure 2. The SY curves of the mixtures of cyclic and linear peptides lie between the SY curves of the pure peptides. The position of the SY curves is related to the relative amount of linear and cyclic peptides [13,27]. The higher the relative amount of cyclic peptide, the closer the SY curve of the mixture is to the SY curve of the cyclic peptide.

ER MS experiments were conducted using caesium-cationised peptides, as our previous observations showed that protonated peptides produce very similar SY curves, limiting their utility. In contrast, alkali-cationised peptides provided better separation between the SY curves of linear and cyclic peptides. Notably, the separation between these curves increased with the size of the alkali metal, with caesium adducts exhibiting the greatest separation and offering the highest sensitivity for quantifying the molar ratio of linear peptides [13]. Additionally, experiments were performed under high trapping gas pressure (3.00 × 10^−5^ mbar) to further enhance the separation of SY curves for cyclic and linear peptides, thereby improving sensitivity. It was observed that higher trapping gas pressure significantly increased the differences between the SY curves [30].

**Figure 4:**
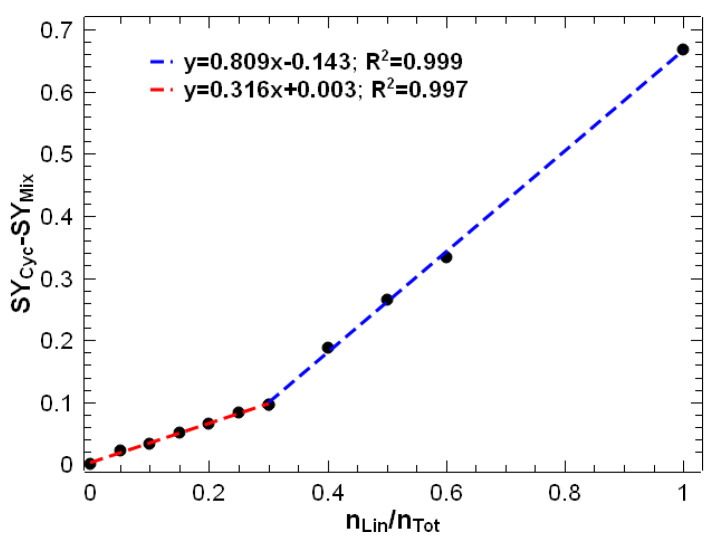
SY at an excitation voltage of 2.2 V for caesium-cationised mixtures of cyclic and linear peptides at different molar ratios. Two regression models were calculated: one for molar ratios of linear peptide from 0 to 0.3 (in red) and another from 0.3 to 1 (in blue).

The difference between the SY of the mixtures and the SY of the cyclic peptide was plotted against the molar ratio of the linear peptide. This plot was made at an excitation voltage of 2.2 V (orange vertical line in Figure 3), as we observed the best results to detect lower amounts of linear peptide under these conditions. Figure 4 shows that two different linear relationships were observed: a linear model for molar ratios of linear peptide between 0 and 0.3 and another linear model with a higher slope for molar ratios from 0.3 to 1. The large difference in sensitivity between the two models is due to matrix effects due to ion suppression, which are commonly observed in electrospray sources in mass spectrometry [9,42,43]. It can be clearly observed that the ionisation of linear peptides is suppressed by the presence of cyclic peptides. Therefore, for lower proportions of linear peptides, the slope of the calibration model is much lower. Conversely, for linear peptide ratios exceeding 0.3, ion suppression is less pronounced due to the reduced relative abundance of cyclic peptide. This is evidenced by the slope of the calibration curve, which is approximately 2.5 times higher than for lower ratios, indicating that sensitivity is diminished in the low range of linear peptide contamination.

**Figure 5:**
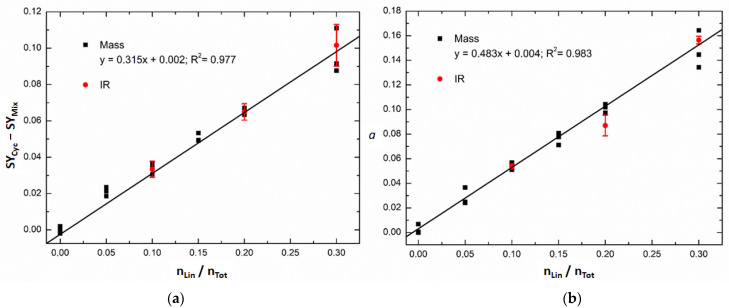
Calibration models of SY data with three replicates (black squares) for mixtures of caesium-cationised linear and cyclic peptides obtained with (**a**) univariate calibration at an excitation voltage of 2.2 V and with (**b**) multivariate calibration (coefficient (*a*) in Equation (2)). The IR samples (red circles) correspond to mixtures initially prepared for IR microscopy and measured by ER MS. The red circles correspond to the mean value of three measurements, and the error bar corresponds to the standard deviation of the three measurements.

Univariate and multivariate calibration models were calculated for the lower interval of linear peptide molar ratios (i.e., from 0 to 0.3) because the aim of this study is to detect the presence of small traces of linear precursor in samples of cyclic peptide. For this range, we measured the SY of each mixture three times. For comparative purposes, the linear peptide molar ratios of the mixtures were the same as the ones measured by IR microscopy: 0, 0.05, 0.1, 0.15, 0.20 and 0.30. Figure 5a shows the univariate calibration model at an excitation voltage of 2.2 V (orange vertical line in Figure 3). This regression model was obtained by calculating at 2.2 V the difference between the SY of the cyclic peptide and the SY of each calibration standard and by plotting this difference against the linear peptide molar ratio of the calibration standards.

Figure 5b shows the results obtained by multivariate calibration to quantify the molar ratio of linear peptides by applying the Classical Least Squares (CLS) algorithm. In this case, instead of using the SY at only one excitation voltage, the SY curve as a whole was used. The SY curve of a calibration standard can indeed be described as a linear combination of the SY curves of caesium-cationised linear and cyclic peptides obtained from pure samples [13,27]. A linear combination coefficient (*a*) can then be obtained, which minimises the sum of squares of residuals (**e**) of the following expression:

(2) $$SY_{\mathrm{Mixture}}=a\times \;SY_{\mathrm{Linear}}+(1-a)\;.SY_{\mathrm{Cyclic}}+e$$

where **SY**_Linear_ and **SY**_Cyclic_ correspond, respectively, to the SY curves of the caesium-cationised linear and cyclic peptides obtained from pure samples (bolded variables describe vectors). Linear coefficients (*a*) obtained for each calibration standard were then plotted against the molar ratio of linear peptide to calculate a univariate regression curve. In this way, the linear coefficient obtained by CLS can be related to the molar ratio of linear peptides of the calibration samples. In the absence of matrix effects due to ionisation efficiency, the slope of this curve should be close to 1. In this case, the slope is only 0.483, much lower than 1. This is because there is ionisation suppression of the linear peptide in the presence of the cyclic peptide (as was already observed in Figure 4 for the interval 0–0.3 of linear peptide molar ratio).

Both calibration models showed good coefficients of determination, R^2^, with the multivariate model being slightly better. Three of the calibration standards initially prepared to be measured by IR microscopy (at molar ratios of linear peptide of 0.1, 0.2 and 0.3) were also measured by ER MS (red circles in Figure 5). The IR standards were diluted 100 times in water/methanol (1:1), and CsCl was added at 180 μM in order to be in the same conditions as the calibration standards used for the ER MS calibration models (black squares in Figure 5). The SY values and the CLS coefficients (*a*) obtained for the IR samples are very similar to the ones obtained for the MS samples. These results confirm that the ER MS measurements are reproducible and that the IR calibration standards were correctly prepared.

The limit of detection, LD, was then calculated by using the information from the calibration model and the following formula:

(3) $$LD=3.3\cdot \frac{s_{e}}{b_{1}}$$

where *s_e_* corresponds to the standard deviation of the residuals of the calibration line (defined also as standard error) and *b*_1_ to its slope. This approach is recommended in LC-MS methods, as it gives conservative estimates when the LD is calculated from calibration graphs [43,44]. The factor of 3.3 is associated with a probability of a false positive decision (type I or α-error) of 0.05 and a probability of a false negative decision (type II or β-error) of 0.05 [45,46]. The LD calculated for the univariate model was a 0.053 molar ratio of linear peptides. The LD calculated for the multivariate model was 0.045, slightly better than the univariate model.

The performance of both calibration models was evaluated by calculating the fit error of each calibration standard as the difference between the predicted value of the linear peptide molar ratio, (*n*_Lin/_/*n*_Total_)_predicted_, and the reference value of the linear peptide molar ratio, (*n*_Lin/_/*n*_Total_)_reference_. Figure S1a shows the fit error of each calibration standard calculated for the univariate and multivariate calibration models of the SY data. All fit errors were randomly distributed around 0 and less than 0.02 (except for the standard at 0.3). Figure S1b shows the percentage of fit error for each calibration standard. Both models show similar values of fit error. The percentage of fit errors was less than 20% except for the standard at 0.05 molar ratio of linear peptide.

The root-mean-square-error of calibration (RMSEC) was calculated to obtain an average fit error for each calibration model:

(4) $$RMSEC=\sqrt{\frac{\sum_{i=1}^{n}{Fit\;error}_{i}^{2}}{n}}$$

where *n* corresponds to the number of calibration standards, i.e., 6 × 3 = 18. The RMSEC values calculated for both models were quite similar and slightly better for the multivariate model: For the univariate model, it was 0.015, and for the multivariate model, it was 0.013.

The intermediate precision was calculated as the pooled variance of all three replicates measured for each calibration standard [46]. The intermediate precision (expressed as standard deviation) was 1.71 × 10^−2^ for the univariate model and 1.58 × 10^−2^ for the CLS, showing that precision is slightly improved when the entire SY curve is used to calculate the molar ratio of linear peptides. Although the performance of the linear and multivariate calibration models is quite similar, the multivariate model shows slightly better performance in terms of limit of detection, RMSEC and intermediate precision. However, it is important to note that the multivariate model requires the measurement of the entire SY curve and is more time-consuming than the univariate model, which only requires the measurement at one excitation voltage.

### 3.2. Mid-Infrared Microscopy

**Figure 6:**
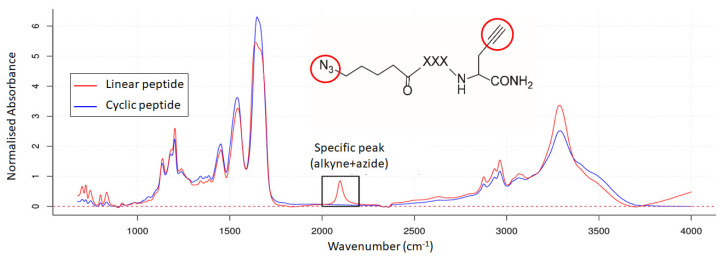
IR spectra of the linear peptide (in red) and the cyclic peptide (in blue). A specific peak is observed for the linear peptide. The red circles indicate the alkyne and azide groups in the linear peptide, which are responsible for this peak.

The pure linear and cyclic peptides were measured by IR microscopy. Figure 6 shows the superposition of the two IR spectra. Each spectrum was normalised to the mean absorbance and baseline corrected. Both spectra show the same absorption bands except for a wavelength region from 2040 to 2170 cm^−1^, which is observed only for the linear peptide. In fact, this band is characteristic of the linear peptide since it corresponds to the alkyne (C≡C) and/or azide (N_3_) functions, which usually appear between 2100–2140 cm^−1^ and 2100–2200 cm^−1^, respectively. The inability to observe the IR peaks of the terminal alkyne and azide separately is due to the limited resolution of the mid-IR spectrum (4 cm^−1^) and the overlapping wavenumber ranges of their characteristic absorption bands.

**Figure 7:**
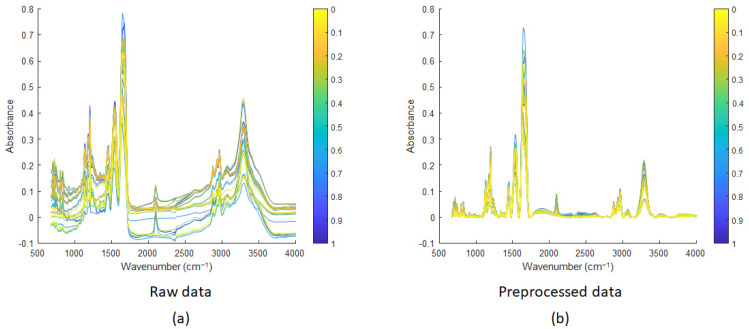
IR spectra of mixtures of linear and cyclic peptides: (**a**) raw data and (**b**) preprocessed spectra with baseline correction using AsLS. The figure uses a colour coding scheme to represent the molar ratios of linear and cyclic peptides. The pure peptides are assigned distinct colours: yellow for the cyclic peptide and blue for the linear peptide. Intermediate colours represent gradients resulting from the proportional mixing of yellow and blue, with each shade corresponding to the specific molar ratio of linear peptide.

Several mixtures of the cyclic and linear peptides were measured by IR microscopy. Each mixture was measured in triplicate. For each triplicate, 100 spectra were measured and averaged (see Section 2.3 of the Experimental section). Figure 7 shows the average spectrum for each triplicate. A total of 33 spectra are shown (i.e., 3 replicates*11 concentration levels). Figure 7a shows that the spectra are baseline shifted. This is usually the case in IR data due to background variations, mainly due to scattering.

Figure 7b shows the IR spectra corrected by Asymmetric Least Squares [47,48]. Asymmetric Least Squares (AsLS) is a spectral baseline correction method that separates the baseline from the signal. AsLS is widely used in spectroscopy and chromatography for accurate baseline correction, effectively distinguishing sharp peaks from smooth baseline components. The advantage of this method is that no prior information about peak shapes or baselines is required. It minimises an objective function combining residuals and a smoothness penalty. Weights are iteratively adjusted: higher for points below the baseline (assumed to be noise) and lower for points above (assumed to be peaks). Parameters include lambda for smoothness and *p* for asymmetry. Both parameters have to be tuned to the data at hand and chosen by data visualisation. In AsLS, *p* (for asymmetry) usually varies between 0.1 and 0.001 and lambda (for smoothness) between 10^2^ to 10^9^ [47]. Different combinations of *p* (0.1, 0.01 and 0.001) and λ (10^3^, 10^4^, 10^5^ and 10^6^) were tried. After visual inspection of the corrected spectra, and avoiding those with negative parts, four combinations were kept with *p*-values of 0.01 and 0.001 and λ values of 10^4^ and 10^5^. The optimal combination (*p* = 0.001 and λ = 1·10^4^) was finally found by cross-validation of the four PLS models and selecting the model with the best predictive performance. In our case, the best baseline correction of the IR spectra was obtained with *p* = 0.001 and λ = 1·10^4^. These values were used to correct the baseline of all the IR spectra. Figure 7b shows that the preprocessed spectra were successfully baseline-corrected.

**Figure 8:**
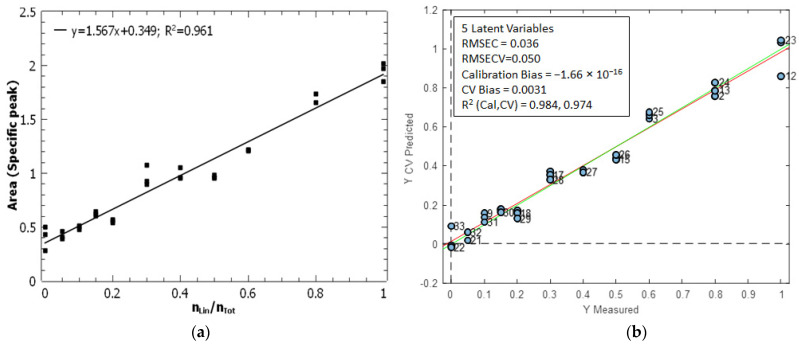
Calibration models to quantify the molar ratio of linear peptides by IR microscopy. (**a**) Univariate calibration is based on the area of the specific peak of linear peptide (Figure 7). (**b**) PLS calibration model calculated from the whole IR spectra. The green line represents the theoretical line *y* = *x*, while the red line represents the regression line derived from the predicted values of the calibration standards.

The molar ratio of linear peptides was quantified through univariate and multivariate calibration, as illustrated in Figure 8. The univariate calibration model (Figure 8a) corresponds to the area of the specific peak against the molar ratio of the linear peptide. A high determination coefficient (R^2^ = 0.961) was observed for the univariate model, indicating a strong correlation between the two variables.

For the multivariate model, the PLS (Partial Least Squares) regression method was applied between the matrix **X** (33 × 1725) containing the IR spectra and the vector **y** (33 × 1) containing the molar ratio of the linear peptide. The model was validated using contiguous-block cross-validation, with 11 data splits (three samples per split). The optimal PLS model had five latent variables and had quite good figures of merit (Figure 8b), with an RMSEC = 0.036 and a root-mean-square-error of cross-validation, RMSECV = 0.050. The model had practically no bias, and the coefficients of determination, R^2^, for calibration and validation were 0.987 and 0.974, respectively.

The RMSEC of the univariate model was of 0.062, while the RMSEC of the PLS model was of 0.036. The PLS model displays superior predictive capabilities in comparison to the univariate model. This is clearly observed in Figure S2a, which shows that the fit errors of the univariate model are higher than those of the PLS model. Figure S2b also demonstrates that the PLS model is more predictive in terms of the percentage of fit error. As for ER MS, the intermediate precision was calculated as the pooled variance of all three replicates measured for each calibration standard [46]. The intermediate precision (expressed as standard deviation) was 5.06 × 10^−2^ for the univariate model and 2.63 × 10^−2^ for the PLS model. This shows that precision is clearly improved when the entire IR spectra are used to calculate the molar ratio of linear peptides. The limit of detection of the univariate model (calculated with Equation (3)) was 0.21. The limit of detection of the multivariate model was calculated as 3.3·RMSEC, which corresponds in multivariate calibration to the equivalent expression of Equation (3). The limit of detection was 0.12, which is considerably lower than that of the univariate calibration. The PLS model demonstrates superior performance in terms of limit of detection, RMSEC and intermediate precision. It is therefore preferable to consider the full IR spectrum rather than focusing on the specific peak of the linear peptide.

### 3.3. Comparison of Energy-Resolved Mass Spectrometry and Mid-Infrared Microscopy

Table 1 presents a summary of the analytical performances obtained for energy-resolved mass spectrometry and mid-infrared microscopy. The results demonstrate that ER MS exhibits superior performance to IR-microscopy in terms of the root-mean-square error of calibration (RMSEC), intermediate precision and the limit of detection. Table 1 demonstrates that the detection limit is significantly lower for ER MS. The univariate model yields a detection limit of 0.053 for ER MS, whereas the detection limit for IR is 0.21, which is approximately four times higher. The detection limit in IR improves for the PLS model. However, even for the multivariate calibration model, the detection limit remains about twice as high as that obtained with ER MS.

One limitation of ER MS is that the calibration models are only linear up to a linear peptide molar ratio of 0.3. The lack of linearity caused by matrix effects can be addressed using an isotopically labelled internal standard. In this approach, the matrix effects on both the analyte and the internal standard are expected to be nearly identical, allowing the internal standard calibration curve to compensate for these effects and improve linearity across a wider range [9,43]. Alternatively, a non-linear calibration model can be employed to account for matrix-induced deviations. In electrospray ionisation (ESI) sources, quadratic models are commonly used to address such non-linearities effectively. To predict samples across the entire working interval, we have calculated a quadratic model using the data, rather than fitting two separate linear models. The results have been included in the Supporting Information (Figures S3 and S4). While the quadratic model provides a unified approach, its performance, in terms of prediction error and RMSEC, was less optimal compared to the two separate linear models. Specifically, the RMSEC for the quadratic model was 0.025, compared to 0.015 for the linear model. Figure S3 shows that the calibration samples with linear ratios of 0.25 and 0.30 exhibited prediction errors of approximately 0.03 and 0.05, respectively. Both values were significantly higher than the errors obtained using the linear model (Figure S1). Therefore, a quadratic model is not suitable for extending the working interval. In contrast, IR-microscopy is linear across the entire working interval, from linear peptide molar ratios of 0 (equivalent to pure cyclic peptide) up to 1 (pure linear peptide). Therefore, energy-resolved mass spectrometry is the optimal choice for the detection and quantification of the smallest amounts of linear peptides. However, infrared microscopy is more suitable for samples with higher molar ratios of linear peptides.

## 4. Conclusions

The successful quantification of mixtures of synthetic linear and cyclic isomeric peptides has been achieved through the utilisation of energy-resolved mass spectrometry (ER MS) and mid-infrared microscopy. The ER MS method was based on univariate and multivariate calibration models, calculated from Survival Yield (SY) data. The multivariate model demonstrated slightly enhanced analytical performance, which can be attributed to more effective signal averaging when SY curves are considered as a whole, resulting in a greater number of data points and more robust statistics. However, the multivariate model requires the entire SY curve to be measured, making it a more time-consuming process than the univariate model, which only requires the measurement of SY at one excitation voltage.

The feasibility of infrared microscopy as an alternative technique for quantifying the relative amount of linear peptide has also been demonstrated. In this case, the Partial Least Squares (PLS) calibration model exhibited superior performance in comparison to univariate calibration, indicating that it is preferable to consider the full IR spectrum rather than focusing on the specific peak of the linear peptide.

The comparative analysis of the two analytical techniques reveals that ER MS demonstrates superior performance compared to IR microscopy. It can therefore be concluded that ER MS is the optimal technique for the detection and quantification of the smallest amounts of linear peptide. However, one limitation of ER MS is that the linearity interval is more restricted than that of IR microscopy. This is due to matrix effects originating in the electrospray source, whereby the ion suppression of the linear peptide in the presence of cyclic peptide results in a restricted linearity interval. In contrast, IR microscopy is linear across the entire working interval, from linear peptide molar ratios of 0 (equivalent to pure cyclic peptide) up to 1 (pure linear peptide). Consequently, infrared microscopy is more appropriate for samples exhibiting higher molar ratios and greater variability in the relative concentration values of linear peptides.

## Figures and Tables

**Table 1:** Analytical performance of univariate and multivariate calibration models for the quantification of the molar ratio of linear peptide by energy-resolved mass spectrometry (ER MS) and IR microscopy.

Analytical Technique	Calibration Model	Linearity Interval	R^2^	Fit Error (RMSEC)	Intermediate Precision	Detection Limit (LD)
ER MS	Univariate	0–0.3	0.977	0.015	1.71 × 10^−2^	0.053
	Multivariate (CLS)	0–0.3	0.983	0.013	1.58 × 10^−2^	0.045
IR microscopy	Univariate	0–1	0.961	0.062	5.06 × 10^−2^	0.21
	Multivariate (PLS)	0–1	0.987	0.036	2.63 × 10^−2^	0.11

## Data Availability

The data presented in this study are contained within the article.

## Supplementary Materials

The following supporting information can be downloaded at: https://www.mdpi.com/article/10.3390/mps7060097/s1, Figure S1: Performance of the univariate and multivariate calibration models of the SY data; Figure S2: Performance of the univariate and PLS calibration models of the IR spectra; Figure S3: Quadratic model calculated for the SY data; Figure S4: Fit error obtained for the calibration samples of Figure S3 predicted with the quadratic model.
