# Supplementary material for: Energy-Resolved Mass Spectrometry and Mid-Infrared Spectroscopy for Purity Assessment of a Synthetic Peptide Cyclised by Intramolecular Huisgen Click Chemistry
Source: Methods Protoc. 2024 Dec 2;7(6):97. doi: 10.3390/mps7060097 (PMC11676744; doi:10.3390/mps7060097)
Supplement: Supplementary file 1 [file mps-07-00097-s001.zip › mps-3251303-supplementary.pdf]

Supporting information

Energy-Resolved Mass Spectrometry and Mid-Infrared Spectroscopy for Purity Assessment of a Synthetic Peptide Cyclised by Intramolecular Huisgen Click Chemistry

Alicia Maroto [1], Ricard Boqué [2], Dany Jeanne Dit Fouque [1] and Antony Memboeuf [1,*]

[1] Univ Brest, CEMCA, CNRS, UMR 6521, 29238 Brest, France
[2] Department of Analytical Chemistry and Organic Chemistry, Universitat Rovira i Virgili, C/Marcel·lí Domingo 1, 43007 Tarragona, Spain
* Correspondence: antony.memboeuf@univ-brest.fr; Tel.: +33-(0)2-98-01-61

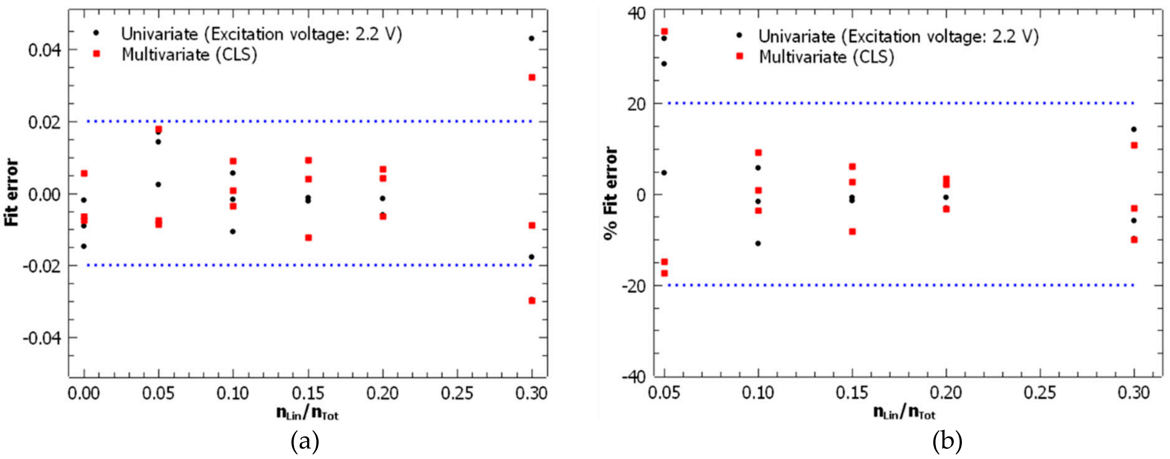

Figure S1. Performance of the univariate (black circles) and multivariate calibration models (red squares) for the SY data, evaluated in terms of (a) fit error and (b) percentage of fit error.

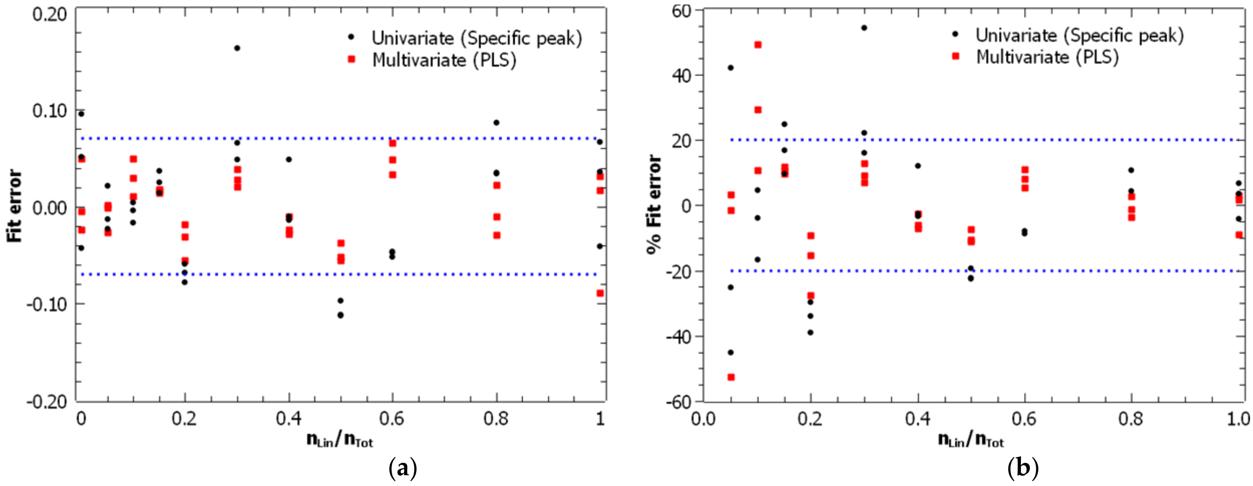

Figure S2. Performance of the univariate (black circles) and PLS (red squares) calibration models for the IR spectra, evaluated in terms of (a) fit error and (b) percentage of fit error.

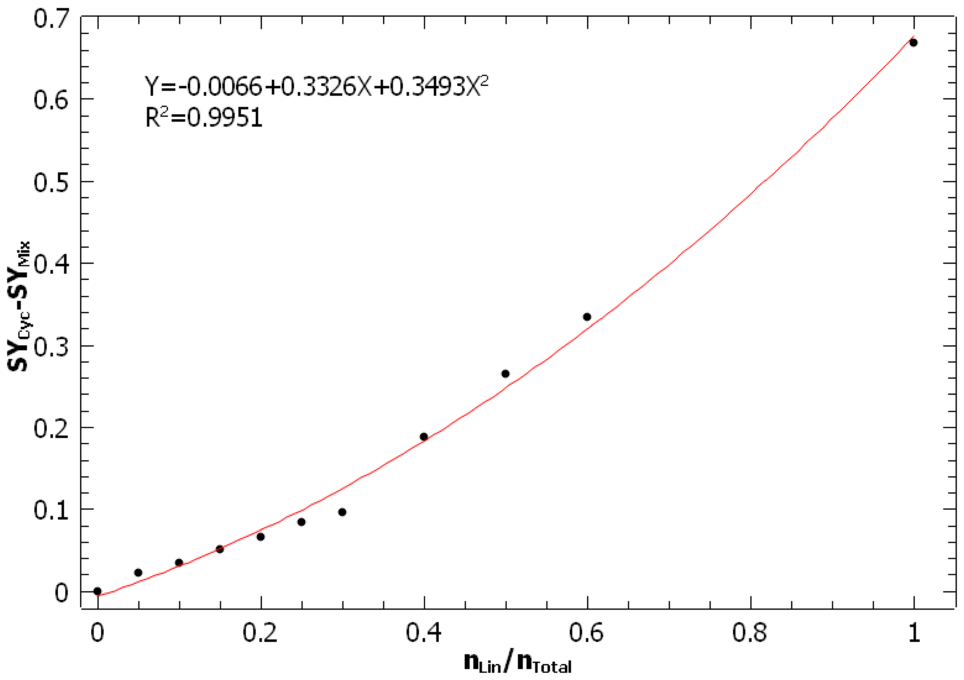

Figure S3. Quadratic model calculated for the SY data (excitation voltage of 2.2 V) of the mixtures of cyclic and linear peptides. The same data are shown in Fig. 4 with two separate linear models.

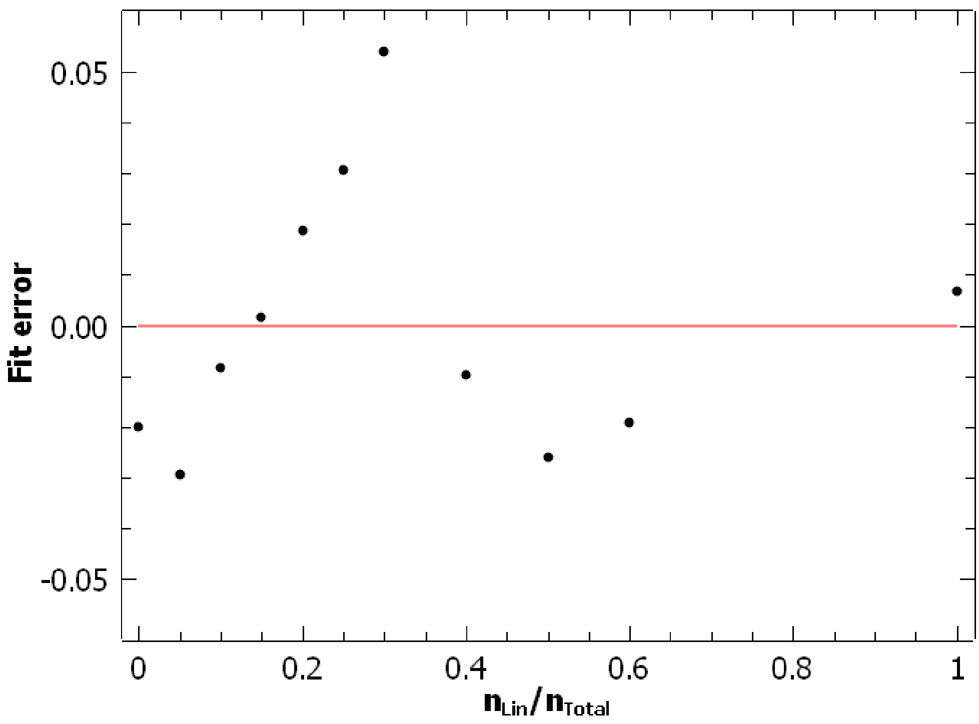

Figure S4. Fit error obtained for the calibration samples of Fig. S3 predicted with the quadratic model.
